# Competitiveness measurement system in the advertising sector

**DOI:** 10.1186/2193-1801-2-438

**Published:** 2013-09-05

**Authors:** Rocío Poveda-Bautista, Mónica García-Melón, Doris C Baptista

**Affiliations:** Departamento de Proyectos de Ingeniería, Universitat Politècnica de València, Camino de vera s/n, Valencia, 46022 Spain; Ciudad Politécnica de la Innovación, Universitat Politècnica de València, INGENIO (CSIC-UPV), Camino de vera s/n, Valencia, 46022 Spain; Departamento de Procesos y Sistemas, Universidad Metropolitana de Caracas, Caracas 1071, Edo Miranda, Venezuela

**Keywords:** Competitiveness measurement system, Measurement indicators, Advertising, Innovation, Analytic Network Process (ANP)

## Abstract

In this paper a new approach to find indicators that can be used to measure companies’ competitiveness and performance in an efficient and reliable way is presented.

The aim is to assist managers of companies within a specific industrial sector by providing information about their relative position in the market so as to define better action plans that may improve the company’s performance.

The approach combines the use of the Analytic Network Process, a multicriteria decision method, with the Balanced Scorecard. It allows the definition of a number of competitiveness indicators based on the performance and setting of the advertising sector. In this way it is possible to obtain a Competitiveness Index that allows a company to know its relative position with respect to other companies in the sector, and establish a ranking of the companies ordered by their competitiveness level.

A case study in the advertising industry of Venezuela is provided. Results show that improvement plans for the agencies analyzed should promote creativity, innovation and the use of new technologies, as a particular form of innovation. These factors were considered to be the most relevant indicators in the advertising sector.

The participating experts agreed that the methodology is useful and an improvement over current competitiveness assessment methods.

## Introduction

Business services companies offer high added-value professional, creative or financial services to other client companies (Taylor 2006).

Advertising is a business service and also a cultural or creative media industry producing aesthetic expressions and sign values (Hermelin 2009) that are communicated through different media formats. The industries of this sector are characterized by being highly customized and differentiated, and creativity is the most essential component of their production (Boojihawon 2007).

The advertising sector operates with two different kinds of organizational structures: Holding Companies/Groups that do the strategic managerial work and Advertising Agencies that provide creative design producer services. This diversification in the services provided responds to competitive reasons. A company cannot provide services to different clients who are competing with each other, but the choice of a certain client might involve losing other strategic clients, which could negatively affect competitiveness. The solution is to create Holding Companies/Groups that constitute a collection of Advertising Agencies (Taylor 2006).

Most of the studies conducted on this sector focus on Advertising Agencies because they provide creativity and innovation into their company clients: Advertising agencies possess greater knowledge about creative work than about market development.

According to Pratt (2006) many authors from different fields of knowledge, such as management, economic growth and innovation, have conducted studies on the notion of the competitive advantage of knowledge, creativity and innovation in the field of advertising (Jeffcutt and Pratt 2002; Lampel et al. 2000; Bell 1992; Taylor et al. 1996).

The advertising industry is experiencing a rapid change and agencies must redefine their roles and adapt to this new competitive environment in order to be better positioned in the market. It is therefore very important for advertising companies to be able to measure competitiveness and relative position in the market.

This new competitive situation comes at a time when Innovation Management and the New Technologies are having a significant impact on the media environment (Durkin and Lawlor 2001). On one hand, studies on the importance of innovation management as a fundamental tool of competitive strategy in service sector companies (Lightfoot and Gebauer 2011; Hertog et al. 2010) and particularly in the communications industry (Ko and Lu 2010). On the other hand, the impact of the new technologies, particularly social networks, on service development has been analyzed in other services sectors such as the hospitality and tourism sectors (Sigala 2012) but not in the advertising sector.

## Measuring competitiveness

In the past, business competitiveness assessment depended only on financial indicators. But recently it also includes factors such as innovation, learning and entrepreneurial capabilities as well as management indicators. Hult et al. (2003) argued that four culture-based factors -entrepreneurship, innovativeness, market orientation and organizational learning- collectively give rise to organizational culture competitiveness.

Porter (1997) defines competitiveness as the ability of a business to systematically maintain the differentiating advantages that allow it to reach, sustain and improve a given socioeconomic position. Porter’s approach assumes that every company that competes in an industrial sector possesses a competitive strategy. This strategy can be developed explicitly through a process of strategic planning or implicitly through the aggregated activity of the different functions of the company. Based on this second assumption, the value chain can be defined as a conceptual structure that helps to diagnose the sources of competitive advantage. In this sense business analysis is based on the analysis of the company’s value chain.

On the other hand, authors like Thakkar et al. (2007) propose the use of Management Systems as analysis tools that help relate the company’s competitive strategy to its performance indicators. These authors consider that the *Balanced Scorecard* model (BSC) (Kaplan and Norton 2000) provides an appropriate framework to translate the company’s strategic objectives to a set of coherent performance indicators. The main advantage of BSC is the close relationship between the clusters of the company’s strategic indicators and the different internal and external, financial and non-financial indicators. Öztayşi and Ucal (2009) consider BSC to be the most suitable technique for measuring business performance due to its great success in the professional and academic world for the alignment of competitiveness indicators with business objectives.

Previous attempts to identify sources of a company’s competitive advantage have concentrated mainly on different manufacturing industries, Liedtka (2005), Jalali Naini (2010) and Grigoroudis, Orfanoudaki and Zopounidis (2012) among others.

According to Hipp and Grupp (2005), the large differences between these industries and services industries limit the application of the findings to the services sector. Indicators related to quality, productivity, raw materials among others (Liedtka 2005; Jalali Naini 2010 and Grigoroudis et al. 2012), which are typically related to competitiveness in manufacturing industries, are difficult to measure in industries related to services. Competitiveness in the services sector should be measured using intangible indicators. Few related approaches have been found in the literature and they all focus on a particular sector but cannot be generalized to the whole service sector (Roy 2011). In the particular case of the advertising industry there are studies that measure competitive performance (Nachum 1996) although these studies do not provide a reliable approach applicable to the services sector. Similarly, no competitiveness studies of the services sector in Venezuela have been found in the literature.

In this paper, we propose a general method for measuring competitiveness, which can be adapted to different specific sectors. The specificity of this approach is based on the experts’ knowledge. In this case, experience and knowledge are the key issues of the problem. Therefore, it is preferable to focus the efforts on finding a renowned group of experts and get them involved in the process.

Since the proposed methodology should measure multifaceted performance characteristics, some of which are intangible, our approach will be based on multicriteria techniques, Multicrieria Decision Analysis (MCDA), in particular on the Analytic Network Process (ANP).

This methodology has been applied to and validated in the sector of the plastic industry (Poveda-Bautista et al. 2012). In the present paper the method is applied to the services sector, in particular to the advertising industry, which contributes 11 percent to Venezuelan GDP.

The rest of the paper is organized as follows: Section “The use of MCDA techniques for competitiveness assessment” reviews the applications of MCDA techniques to the field of this research; in Section “Methodology” the methodology of the study is proposed; Section “Case study” develops the case study; and the last sections present analysis and discussion of the results obtained and some conclusions.

## The use of MCDA techniques for competitiveness assessment

Competitiveness assessment is a challenging task aiming at enhancing performance in the context of continuous improvement. To accomplish this task, companies must have an organizational system based on analytical models designed to measure multifaceted performance (Augusto et al. 2008). MCDA techniques are very suitable for solving this type of problems. The expression MCDA is used as an umbrella term to describe a number of formal approaches which take explicit account of multiple criteria and helping individuals or groups explore decisions that matter (Belton and Stewart 2002). More information about MCDA can be found in Belton and Stewart (2002) and Barba-Romero and Pomerol (1997).

Several authors have recently introduced the use of MCDA techniques for measuring companies’ competitiveness as well as for academic competitiveness measure (Ren and Gong 2012), (Ding and Qiu 2010). Most of these studies focus on building decision models and developing decision-making methods. Many of them use the Analytic Hierarchy Process (Saaty 1996) which has been accepted as a leading multi-criteria decision model (Shahin and Mahbod 2007), (Sirikrai and Tang 2006), (Temur et al. 2007), (Reisinger et al. 2003), (Lee et al. 2008a) to assign priorities to the criteria or indicators involved in a decision process, and the last two authors also apply Balanced Scorecard to performance measures. This MCDA technique works well under the assumption of the independence of criteria. However, this assumption is not always realistic, particularly in the field of competitiveness measurement where multiple related dimensions of information have to be considered in the analysis. For this reason, in the model used in the present work is based on the Analytic Network Process (ANP) as it takes into account the interdependence among criteria.

The Analytic Network Process (ANP) is a method proposed by Saaty. It provides a framework for dealing with decision-making or evaluation problems. It presents its strengths when working in scenarios with scarce information, like is the case of competitiveness measurement. ANP is based on deriving ratio-scale values to be used to allocate resources according to their ratio-scale priorities; ratio-scale assessments, in turn, enable considerations based on trade-offs (Keeney and Raiffa 1976). ANP models the process using a network of criteria and alternatives (all called elements), grouped into clusters. All the elements in the network can be related in any possible way. This provides an accurate modeling of complex settings and allows handling the usual situation of interdependence among elements for company evaluation and assessment.

In the literature some applications have been reported involving ANP in the field of competitiveness; some works are related to companies performance measures (Lin et al. 2009), (Yang et al. 2009); others to strategic e-business decision analysis (Raisinghani et al., 2007) and others to Customer Relationship Management in e-commerce companies (Öztayşi et al. 2011).

Some of these applications combine BSC and ANP and also use fuzzy logic. In these cases the four BSC perspectives are used as a framework to measure companies’ competitiveness performance using financial as well as non-financial indicators (Leung et al. 2006), (Yüksel and Dagdeviren 2010), (Tseng 2010) and (Hsu et al. 2011). Specifically, the latter incorporates sustainability indicators to the BSC model. In all of these works the ANP model is composed of clusters grouped according to the four BSC perspectives and their dependence relationship. Other applications based on a combination of BSC with ANP are applied to New Product Development (Lee et al. 2008b). However, to our knowledge, no application has been found based on experts’ judgments aggregation which considers interaction among indicators.

In our proposal only the experts’ opinions and judgments are taken into account as input data in the evaluation model.

### Theoretical background of the ANP model

The Analytic Hierarchy Process (AHP) and the Analytic Network Process (ANP) are two methods proposed by Saaty (Saaty 1980,1996,2000,2005, 2008). AHP is a well-known technique, widely used and conceptually easy to use. However its strict hierarchical structure cannot address the complexities of many real-world problems. As a solution, Saaty proposed the ANP model, a generalization of AHP. ANP represents a decision-making problem as a network of criteria and alternatives (all called elements), grouped into clusters. The influence of the elements in the network on other elements in that network can be represented with a supermatrix. This new concept consists of a two-dimensional element-by-element matrix which adjusts the relative importance weights in individual pairwise comparison matrices to build a new overall supermatrix with the eigenvectors of the adjusted relative importance weights.

According to (Saaty 2001), the ANP model comprises the following steps:

(i.)    Identifying the components and elements of the network and their relationships.
(ii.)    Conducting pairwise comparisons on the elements.
(iii.)    Placing the resulting relative importance weights (eigenvectors) in pairwise comparison matrices within the matrix (unweighted matrix).
(iv.)    Conducting pairwise comparisons on the clusters.
(v.)    Weighting the blocks of the unweighted matrix, by the corresponding priorities of the clusters, so that it can be column-stochastic (weighted matrix).
(vi.)     Raising the weighted matrix to limiting powers until the weights converge and remain stable (limit matrix).
(vii.)     Obtain the elements prioritizations according to any of the columns of the limit matrix.
      The priority of each alternative (company) is a dimensionless value that will be considered the Agency Competitiveness Index (ACI).
(viii.)      Once the results are obtained, in case some alternatives achieve very similar results, a sensitivity analysis should be carried out in order to demonstrate the robustness of the ranking obtained.

## Methodology

The methodology proposed in this study applies industrial competitiveness measurement following the proposal of Ellis et al. (2002), who suggest that measurement indicators depend on the type of industrial sector and the competitiveness level perceived by each sector. This methodology allows the definition of a number of competitiveness indicators based on the performance and setting of an industrial sector, and their implicit relationships. In this way it is possible to obtain a Competitiveness Index that allows a company to know its relative position with respect to other companies in the sector, and establish a ranking of the companies ordered by their competitiveness level within that particular industrial sector.

The methodology has already been applied to the plastic sector (Poveda-Bautista et al. 2012) and demonstrated that it can be very useful for providing insight into the philosophy of what competitiveness in one particular sector is as well as for formulating companies’ improvement plans.

The methodology presented also allows identifying specific indicators of competiveness measurement for the services sector and in particular for the advertising sector.

The methodology is based on three main aspects:

-  The competitiveness model defined by Porter (1995)
-  The BSC system developed by Kaplan and Norton (2000)
-  The multi-criteria ANP model developed by Thomas Saaty (2005) which allows modeling the decision problem as a network of inter-related elements (indicators and companies in our case study).

**Figure 1:**
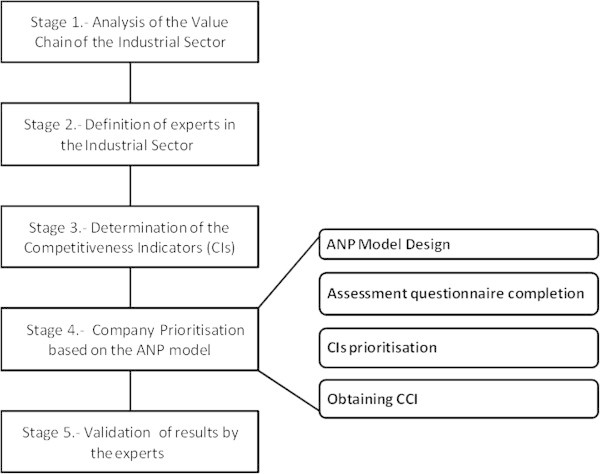
**Proposed methodology.**

Figure 1 shows the five stages of the methodology proposed in this study: A detailed description of each of these steps is presented within the case study.

## Case study

To demonstrate the applicability of the proposed methodology to the advertising sector, it has been used to assess the competitiveness of three Venezuelan agencies.

The aim is to obtain an Agency Competitiveness Index (ACI) for each of them, which will allow comparing their relative position in the sector. However the most important findings will be the specific list of Competitiveness Indicators and their weights for the advertising sector.

Venezuela has the Venezuelan Federation of Advertising Agencies (FEVAP), a business group whose mission is to strengthen the value of advertising and communications in the country. The three selected agencies belong to this federation and have been chosen because they provide services of creativity and innovation into other Venezuelan companies and are located in Caracas. Due to confidential reasons the name of these three agencies will not appear in the paper. They will be called Company A, Company B and Company C.

### Stage 1: Value chain

The analysis of the components of the value chain allows us to define the factors that more strongly affect company competitiveness (Spendolini 1994). The components of the Value Chain are used to lay the foundations for the competitive performance of a particular industrial sector. The value chain of the advertising sector includes the three following main areas: Media Recruitment, Creative Department and Accounts Department. Media Recruitment refers to advertising media suppliers; the Creative Department includes the service producer team - advertising campaign- and also performs quality control; and, finally, the Accounts Department is responsible for liaising with the end customer.

**Figure 2:**
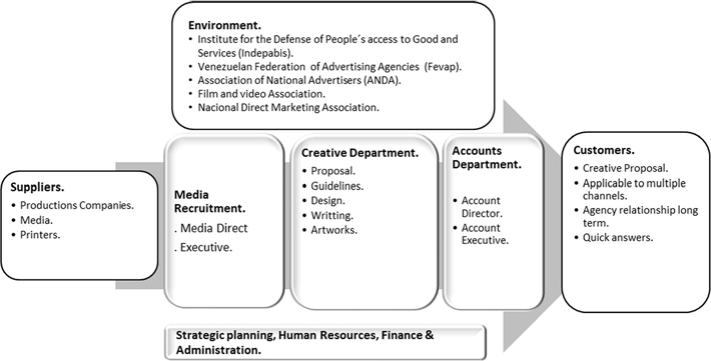
**Value chain of the advertising industry.**

This sector has a close relationship with its clients, as they seek a long-term relationship based on quality, effective response time and innovative proposals. During the construction of the value chain the following factors were identified as critical of this sector: raising agency recognition by their advertisers and international awards for innovation and creativity in advertising campaigns. At the same time, it is important to have long-term accounts department managers and train creative department managers on new advertising techniques. Figure 2 shows the value chain of the advertising industry.

### Stage 2: Selection of experts

When the information available is uncertain it is necessary to make estimates. In such cases, experience and knowledge of the problem are more important than the prioritization technique itself. For the goal of the study -prioritization of agencies and indicators for competitiveness measurement in the advertising industry -the experts selected possess deep knowledge of this industry in Venezuela, management systems and competitiveness levels. Knowing the required profile of the experts, and together with the CEO of Venezuelan Federation of Advertising Agencies (FEVAP), the three experts were chosen:

-  Expert one: Executive vice president, general manager of an advertising agency. It has eighteen years of experience in the sector. He is a member of Fevap’s Board of Directors since 2005. He has been chairing Fevap’s Board for the period 2010–2012. In 2009 he won the award for Best Professional, awarded by the Venezuelan magazine Publicidad and Mercadeo.
-  Expert two: President of an advertising agency. Second vice president of Fevap for the period 2008–2010.
-  Expert three: President of an advertising agency. Degree in Advertising and Marketing. Fevap President for the period 2008–2010.

### Stage 3: Competitiveness indicators

At this stage, and based on the value chain, the experts select the indicators that better represent the expectations of the sector and the factors that more significantly affect the agencies’ competitive performance. A total of 15 indicators were finally selected and grouped according to the four BSC aspects.

In the first meeting the experts discussed the key areas of the value chain, particularly Media Recruitment, Creative Department and Accounts Department and the Competitiveness Indicators (CIs) associated with them.

In the second meeting the experts were asked to reduce the number of indicators by consensus. After a two-hour discussion, a total number of 15 relevant indicators were selected. The four aspects of Kaplan and Norton’s BSC model (2000) were used as analysis framework to help experts in the definition of the indicators.

The competitiveness indicators selected were:

A. Customers:
  1. Market share: Customer yearly turnover/Agency total turnover.
  2. Rate of new customers: New customers in the year/Total customer base.
  3. Customer Loyalty: Average age of the customers/ Year life of the company.
  4. Response time: Average time to respond in days.
B. Finance: we selected the most relevant financial indicators in financial management and competitiveness.
  1. Indebtedness: Total debts/Heritage.
  2. Liquidity: (Active- Stock)/Passive. It must be greater than one.
  3. Return of equity: Net utility before of taxes/Heritage.
C. Internal process: this component refers to the identified critical processes relative to competitiveness.
  1. Awards: Number of awards won annually/Annual nominations.
  2. Environmental and social responsibility: Environmental and social responsibility implemented measures/Proposal measures.
  3. New services capability: measured with a 1-9 scale: 1 non-influential – 9 highly influential.
  4. New technology use: Staff using new technologies/Total staff.
D. Learning and growth
  1. Creative capability: measured with a 1-9 scale: 1 non-influential– 9 highly influential.
  2. Successful proposal: Successful proposal/Total Proposal.
  3. Retention of accounts managers: Number of accounts managers that work for more than two years in the company/Total number of accounts managers.
  4. Strategic decision making: measured with a 1-9 scale: 1 non-influential– 9 highly influential.

### Stage 4: Company prioritization based on the ANP model. Agency competitiveness index

The aim of this step is to obtain an index for each company which indicates the level of competitiveness according to all the indicators considered, Agency Competitiveness Index (ACI). The higher the value of the index the more competitive the agency is.

**Figure 3:**
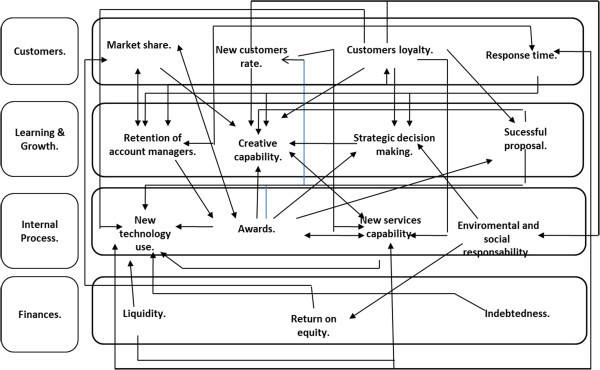
**Diagram of the influences among competitiveness indicators according to the advertising sector value chain.**

This stage covers the following steps:

4.1  Definition of the ANP model. Five components or clusters are built up. The first four correspond to competitiveness indicators, CIs (ANP criteria) grouped according to the four BSC factors. The fifth component corresponds to the companies in the industrial sector, advertising agencies (ANP alternatives).
   The company is a system composed of interrelated subsystems. The indicators measure the competitive performance of the subsystems; therefore, if the subsystems have some kind of influence on each other, the competitive indicators will too.
4.2  With the help of the experts the influence of each element of the model on the others (indicators and agencies) is determined using Saaty’s scale (Saaty 1980) for the pairwise comparisons through questionnaires specifically designed for that purpose. Individual questionnaires were designed and emailed to each of the experts using pairwise questions in order to allow comparison analysis. The group of experts identified the relationships or influences among the 15 indicators. Their results are shown in Figure 3.

Figure 3 reveals a feedback among the components Customers, Internal Process, and Learning and Growth. On the other hand, experts also identified influences among the elements of the four components, for example: *Creative capability* has influence over *Customers loyalty*, which in turn has influence over *New technology use*, which in turn has influence over *Liquidity*.

These relationships are used to build the ANP model for the case study, including a fifth component that contains three advertising agencies belonging to different holding companies/groups whose competitiveness level is to be evaluated. Feedback among the selected agencies is neglected. Due to confidentiality reasons the agencies are referred to as A, B and C.

These agencies were selected from the ones belonging to FEVAP, leaders and competitors among them in the field of advertising. They also had to use similar marketing techniques. In short, these companies should be comparable to each other and clearly identified in the value chain built for the sector.

4.3  The individual experts’ judgments were processed for the calculations. The individual judgments obtained through questionnaires were aggregated using the geometric mean (Saaty, 1980).

**Figure 4:**
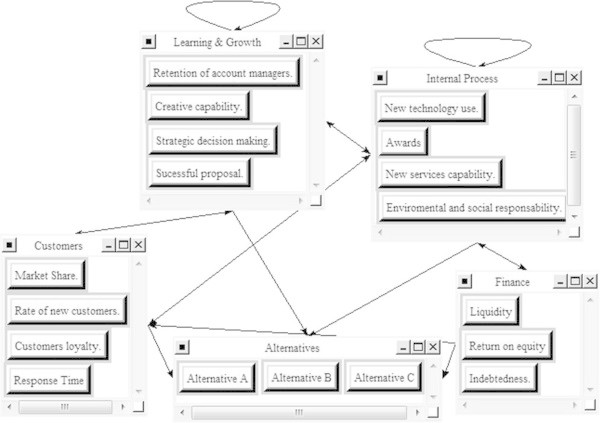
**Diagram of the ANP model.**

With the help of Super Decisions© software (Superdecisions 2009), a software program that performs matrix calculations, the model that represents the ANP-based diagram of influences shown in Figure 4 was developed.

The Influences Matrix was built up based on the relationships shown in Figure 4. This matrix transforms the influential relationships of Figure 3 into a matrix with values 0 and 1 (see Table 1).

**Table 1:** **Influences matrix**

			Companies.			Customers.				Finances.			Internal process.				Learning and growth.			
			A	B	C	C1.1	C1.2	C1.3	C1.4	C2.1	C2.2	C2.3	C3.1	C3.2	C3.3	C3.4	C4.1	C4.2	C4.3	C4.4
Companies.	A	Company A.	0	0	0	1	1	1	1	1	1	1	1	1	1	1	1	1	1	1
	B	Company B.	0	0	0	1	1	1	1	1	1	1	1	1	1	1	1	1	1	1
		Company C.	0	0	0	1	1	1	1	1	1	1	1	1	1	1	1	1	1	1
Customers.	C1.1	Market share.	0	0	0	0	0	0	0	0	1	0	0	1	0	0	1	0	0	0
	C1.2	Rate of new customers.	0	0	0	0	0	0	0	0	0	0	0	1	0	0	0	0	0	0
	C1.3	Customers loyalty.	0	0	0	0	0	0	0	0	0	0	0	1	0	0	1	0	0	0
	C1.4	Response time.	0	0	0	0	0	0	0	0	0	0	0	0	0	0	1	0	0	0
Finances.	C2.1	Indebtedness.	0	0	0	0	0	0	0	0	0	0	0	0	0	0	0	0	0	0
	C2.2	Liquidity.	0	0	0	0	0	0	0	0	0	0	0	0	0	1	0	0	0	0
	C2.3	Return on equity.	0	0	0	0	0	0	0	0	0	0	0	0	0	0	0	0	0	0
Internal process.	C3.1	Awards.	0	0	0	0	0	1	1	1	0	1	0	1	1	0	0	0	0	1
	C3.2	Environmental and social responsibility.	0	0	0	1	0	0	0	0	0	0	0	0	1	0	1	0	0	0
	C3.3	New services capability.	0	0	0	0	1	1	0	1	0	0	0	10	0	0	0	1	0	0
	C3.4	New technology use.	0	0	0	0	1	1	0	0	0	0	0	0	0	0	0	0	0	0
Learning and growth.	C4.1	Creative capability.	0	0	0	1	0	1	1	0	0	0	0	0	0	0	0	0	0	0
	C4.2	Successful proposal.	0	0	0	1	1	1	1	0	0	0	0	1	1	0	0	0	1	1
	C4.3	Retention of account manager.	0	0	0	0	0	1	1	0	0	0	0	1	0	1	0	0	0	0
	C4.4	Strategic decision making.	0	0	0	0	0	1	0	0	0	0	0	1	0	0	0	0	0	0

At this stage and through questionnaires answered by the experts, the degree of the influences among the indicators was obtained using Saaty’s 1–9 scale (Saaty 2000).

**Figure 5:**
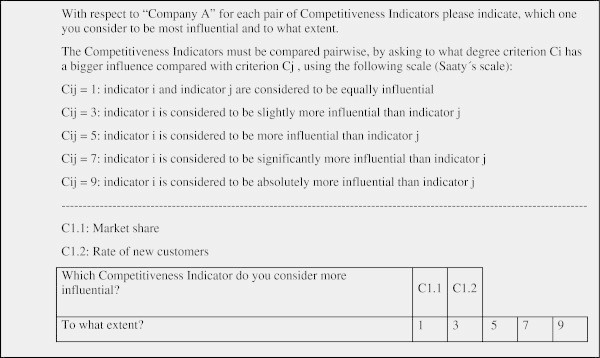
**Sample of questionnaire used for comparison of alternatives.**

An example of the questionnaire used in the comparison analysis is shown in Figure 5:

The Original Supermatrix containing the eigenvalues resulting from the submatrices generated by pairwise comparison of the elements or indicators was modelled based on the geometric mean value of the judgements assessed by each expert. Table 2 shows the values of the resulting Supermatrix.

4.4  With this method a dimensionless value is obtained for each element of the model. The value indicates the importance of the Competitiveness Indicators (CIs) in the evaluation model as well as the relative position of the company in the sector compared to that of the other companies, Agency Competitiveness Index (ACI).

**Table 2:** **Original supermatrix**

			Companies.			Customers.				Finances.			Internal process.				Learning and growth.			
			A	B	C	C1.1	C1.2	C1.3	C1.4	C2.1	C2.2	C2.3	C3.1	C3.2	C3.3	C3.4	C4.1	C4.2	C4.3	C4.4
Companies.	A	Company A.	0	0	0	0.65481	0.10945	0.23848	0.16342	0.24374	0.58763	0.68709	0.77202	0.06949	0.22905	0.14678	0.28506	0.27176	0.28719	0.11841
	B	Company B.	0	0	0	0.24986	0.30900	0.62503	0.29696	0.68709	0.32339	0.24374	0.17344	0.34835	0.69552	0.76922	0.65268	0.66121	0.63485	0.08098
		Company C.	0	0	0	0.09534	0.58155	0.13650	0.53961	0.06917	0.08898	0.06917	0.05455	0.58216	0.07543	0.08400	0.06226	0.06703	0.07796	0.73062
Customers.	C1.1	Market share.	0	0	0	0	0	0	0	0	0	1	0.66667	0	0	0	0	0	0.18673	0
	C1.2	Rate of new customers.	0	0	0	0	0	0	0	0	0	0	0.33333	0	0	0	0	0	0	0
	C1.3	Customers loyalty.	0	0	0	0	0	0	0	0	0	0	0	0	0	0	0	0	0.74289	0
	C1.4	Response time.	0	0	0	0	0	0	0	0	0	0	0	0	0	0	0	0	0.07039	0
Finances.	C2.1	Indebtedness.	0	0	0	0	0	0	0	0	0	0	0	0	0	0	0	0	0	0
	C2.2	Liquidity.	0	0	0	0	0	0	0	0	0	0	0	0	0	0	0	0	0	0
	C2.3	Return on equity.	0	0	0	0	0	0	0	0	0	0	0	1	0	0	0	0	1	0
Internal process.	C3.1	Awards.	0	0	0	1	0	0	0	0	0	0	0	0	0.24998	0	0	0	0	0
	C3.2	Environmental and social responsibility.	0	0	0	0	0.24998	0.15705	0	0	0	0	0	0	0	0	0	0	0	0
	C3.3	New services capability.	0	0	0	0	0.75002	0.24930	1	0	0.33333	0	0.24998	0	0	0	1	0	0	0
	C3.4	New technology use.	0	0	0	0	0	0.59365	0	1	0.66667	0	0.75002	0	0.75002	0	0	1	0	0
Learning and growth.	C4.1	Creative capability.	0	0	0	0.11111	1	0.03690	0.05729	0	0	0	0.06337	0	1	0	0	1	0	1
	C4.2	Successful proposal.	0	0	0	0	0	0.07871	0	0	0	0	0.16695	0	0	0	0	0	0	0
	C4.3	Retention of account manager.	0	0	0	0.88889	0	0.20315	0.18091	0	0	0	0	0	0	0	0	0	0	0
	C4.4	Strategic decision making.	0	0	0	0	0	0.68124	0.76180	0	0	0	0.76969	1	0	0	0	0	0	0

With the weights of the components and the Original Supermatrix the Limit Matrix was constructed raising the weighted matrix to limiting powers until the weights converged and remained stable. In this way, the weights of the 15 indicators and the position of the three companies under study are obtained (Table 3).

**Table 3:** **Results obtained and normalized from the Limit Matrix**

	Element.	Weight.
A	Company A.	0.24581
B	Company B.	0.65276
C	Company C.	0.10143
C1.1	Market share.	0.00262
C1.2	Rate of new customers.	0.00088
C1.3	Customers loyalty.	0.00326
C1.4	Response time.	0.05985
C2.1	Indebtedness.	0.00000
C2.2	Liquidity.	0.00000
C2.3	Return on equity.	0.00002
C3.1	Awards.	0.03227
C3.2	Environmental and social responsibility.	0.00012
C3.3	New services capability.	0.34942
C3.4	New technology use.	0.08676
C4.1	Creative capability.	0.38216
C4.2	Successful proposal.	0.00455
C4.3	Retention of accounts managers.	0.01303
C4.4	Strategic decision making.	0.06505

The results of the prioritization of the three agencies in terms of competitive performance places Agency B in the first position with an ACI of 65.27%, second position for Agency A with an ACI of 24.58%, and third position for Agency C with an ACI of 10.14%.

### Stage 5: Validation of the results by the experts

The results obtained were discussed in order to define patterns that could help in the implementation of plans for improving the company’s competitiveness system.

**Figure 6:**
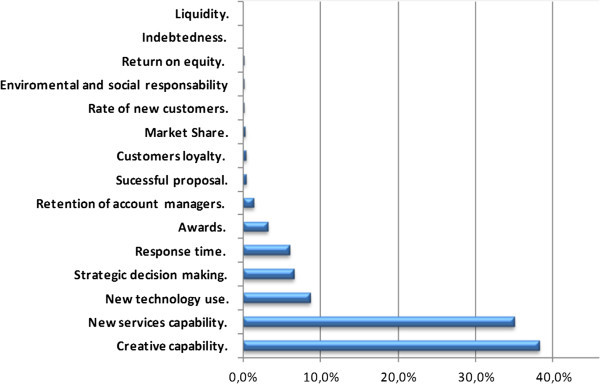
**Weights of the competitiveness indicators.**

Figure 6 summarizes the prioritization of the Competitiveness Indicators provided by the three experts. This figure shows these Indicators sorted out by weight or relative priority.

These global results show that according to the experts the most important criterion is *Creative capability* with 38.21% of the weight, closely followed by *New services capability* (34.94%), *New technology use* (8.67%), *Strategic decision making* (6,5%) and *Response time*(*5*.*98*).

It is also remarkable that according to these findings there are indicators which show weights lower than 0.1%, they are almost negligible. Those are: *liquidity*, *indebtedness*, and *return on equity*, surprisingly within the finances cluster, and *environmental and social responsibility*.

**Figure 7:**
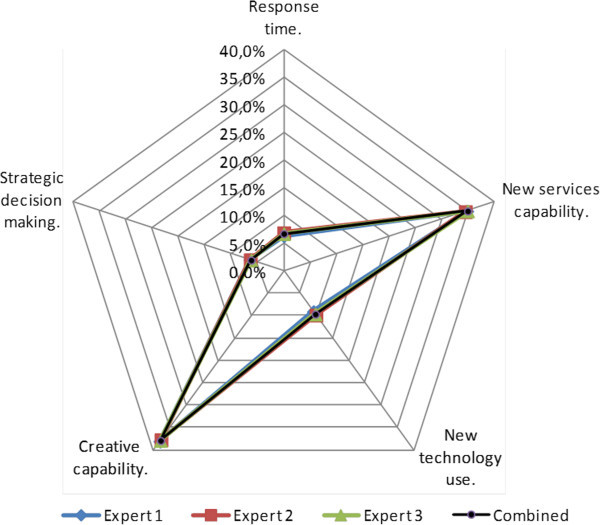
**Analysis of the alignment of the results obtained for the competitiveness indicators.**

**Figure 8:**
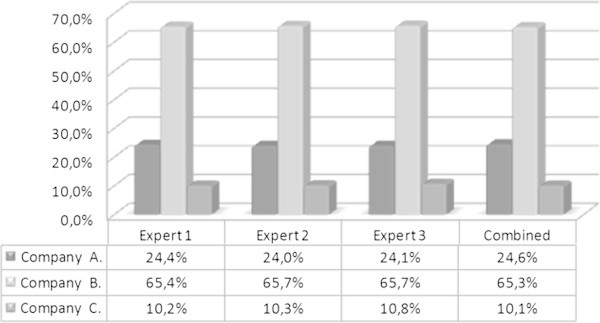
**Comparison of agencies prioritization provided by the experts.**

A further revision of the results provided by each expert reveals that they all agree in the prioritization of the most important indicators and in the relative position of the agencies as can be seen in Figures 7 and 8.

## Results and discussion

The indicators that contribute most to the competitiveness of advertising sector are *Learning and growth*, which highlights the importance of human talent in advertising agencies and, in particular, their creative and innovative capabilities. Second are the indicators relative to internal processes, which explain the need of agencies to innovate not only in the services provided to customers but also in their processes as a form of organizational innovation.

In today’s competitive environment, advertising agencies see the need to foster the creativity of their employees as it helps to develop new ideas in advertising services. Similarly, the ability to present new ideas to customers is a form of innovation as it involves not only new ideas in advertising services but also in organizational practices and procedures.

The use of new technologies also allows customers to participate actively in services innovation and the incorporation of the latest technologies for the development of their advertising services.

In contrast to the importance given in the literature to the agency-client relationship in advertising services, in our study customer-based indicators have a low weight. This is because the indicators belonging to this category, as part of the value chain, relate almost entirely to market ratios and not to services quality as perceived by the client, except for the indicator *Response time*, which has a high weight value.

The economic indicators also have low weights in the measurement system of the sector’s competitiveness. These results are typical in services industries where the added value in the value chain is intangible compared to the tangible products produced by manufacturing industries.

Based on the results, the ACI values of the agencies were reviewed in order to define improvement action plans. Agency C, placed in the third position, got the lowest index value of the three. Therefore the improvement plans for Agency C should focus on the development of strategies that allow it to increase creative capability, new services capability and use of new technologies, i.e. the most important indicators.

In general, for these three agencies, the improvement plans should address creativity, innovation, and the use of new technologies as a specific form of innovation both in services and in customer involvement in new services development. Additionally the organizations should innovate not only in services to customers but also in internal processes to improve response time and strategic decision making.

Finally a satisfaction survey based on Smith-Perera et al. 2010 was conducted on the experts. Based on the results of this survey we can conclude that the method proposed in this paper is useful and seems sufficiently rigorous and reliable. The experts found the results obtained coherent.

The survey is shown in Table 4. The scale used was 1 (lowest) to 5 (highest). The results of the survey indicate that the item about the experts’ satisfaction with the results was scored 4; the item about process efficiency had a score of 4, process complexity was scored 2, and likelihood of using the method in the future had a score of 4.

**Table 4:** **Satisfaction survey** ***conducted on the experts***

*In your opinion*, *the results obtained with the methodology with respect to what you exp*ected are:				
1. Very little satisfactory	2. Little satisfactory	3. Somehow satisfactory	4. Satisfactory	5. Highly satisfactory
In your opinion, the decision-making process used was:				
1. Very inefficient	2. Inefficient	3. Somehow efficient	4. Efficient	5. Very efficient
In your opinion, the process was:				
1. Very difficult	2. Diffícult	3. Normal	4. Easy	5. Very easy
In your opinion, would you use this methodology in the future?				
1. Never	2. Maybe	3. Possibly	4. Most probably	5. Certainly

## Conclusions

The present work describes a new approach based on ANP to assess companies’ competitiveness performance in an efficient and reliable way. It includes an indicator selection process adapted to the particular service sector under analysis. The approach combines the use of the Analytic Network Process (ANP) method with the Balanced Scorecard to define Competitiveness Indicators. The use of ANP can be justified by its ability to obtain quantitative values from experts’ qualitative judgments and also because it enables the aggregation of the experts’ judgments. The experts were chosen based on their experience and knowledge of the advertising sector.

Porter’s BSC paradigm facilitates the understanding of competitiveness as a whole. The analysis of the value chain of companies with similar characteristics in the same sector helps to identify indicators of competitiveness, which will then be prioritized with ANP. For validation the methodology was applied to the advertising industry of Venezuela.

The criteria (indicators) weighting provides some important insights into the overall philosophy and underlying experts’ conception of what competitiveness in the advertising sector is. The resulting data for the indicators show that *Creative capability*, *New services capability* and *New technology use* are the most important indicators to consider when analyzing competitiveness in this sector.

Based on the results we may conclude that:

-  Creative capability: to improve competitiveness advertising agencies should stimulate the creativity of their employees thus contributing to the development of novel and useful ideas about services.
-  New services capability: it includes two forms of innovation, innovation related to creativity in the development of new ideas in advertising services and organizational practices and procedures.
-  Use of New technologies: this involves active customer participation in services innovation processes and the incorporation of new technologies in the development of advertising services.

The results obtained for the different indicators analyzed allow the companies to diagnose their competitiveness performance and develop improvement and innovation plans aligned with these indicators. The improvement plans should focus on creativity, innovation, and the use of new technologies as a specific form of innovation in services and in customer involvement in new services development. Additionally the organizations should innovate not only in services to customers but also in internal processes.

Regarding the results obtained by the three agencies, their ACI values enabled them to analyze their competitiveness performance with respect to other agencies of the advertising sector as well as to compare among them. Agencies used this data to review their situation and to formulate well-defined improvement actions: they should align their strategic objectives with the main objectives obtained with this methodology. Moreover, agencies should invest more resources in creativity, innovation and new technologies.

The experts also showed agreement and satisfaction with the process and the results though they suggested that it was complex and hard, particularly categorizing the influences among the Competitiveness Indicators of the case study.

Based on the review of the literature and the findings of the present study we can conclude that it is not so important for an organization to measure all areas in a competitiveness system since it may become a cumbersome and complex process; by contrast, it is relevant for any organization to have clear goals as well as the metrics and corresponding weights that directly contribute to reach the goals. The ANP model efficiently contributes to define the necessary indicators.

Although both the experts and the decision makers were satisfied with the methodology, the ANP procedure was not free of criticism. During the ANP application to the case study some difficulties arose, such as that ANP prescribes comparisons that occasionally may be complex to understand by experts not familiarized with the method. Hence, careful attention must be devoted to the design of the questionnaires and the comparison process must be helped by a facilitator. Despite these difficulties, the results obtained in this work allow us to conclude that ANP is a suitable tool for assessing the competitive performance of companies. Although the new proposal has been specifically applied to the advertising industry, this tool can be adapted to any services sector, provided the criteria are correctly identified and there are some dependencies among them. This tool opens a very promising future research line in the field of competitiveness measurement systems.

## References

[CR1] Augusto M, Lisboa J, Yasin M, Figueira JR (2008). Benchmarking in a multiple criteria performance context: An application and a conceptual framework. Eur J Oper Res.

[CR2] Barba-Romero S, Pomerol J (1997). Decisiones Multicriterio. Fundamentos Teóricos y Utilización Práctica.

[CR3] Bell JA (1992). Creativity, commercial popularity, and advertising expenditures. International journal of advertising.

[CR4] Belton V, Stewart T (2002). Multiple criteria decision analysis: an integrated approach.

[CR5] Boojihawon D (2007). Network dynamics and the internationalisation process of small advertising agencies. The Service Industries Journal.

[CR6] Ding J, Qiu J (2010). An Approach to Improve the Indicator Weights of Scientific and Technological Competitiveness Evaluation of Chinese Universities. Scientometrics.

[CR7] Durkin M, Lawlor MA (2001). The implications of the Internet on the advertising agency-client relationship. The Service Industries Journal.

[CR8] Ellis S, Elnatha D, Raz T (2002). Applying benchmarking: an organizational learning perspective. Human System Management.

[CR9] Grigoroudis E, Orfanoudaki E, Zopounidis C (2012). Strategic performance measurement in a healthcare organisation: A multiple criteria approach based on balanced scorecard. Omega.

[CR10] Hermelin B (2009). Producer service firms in globalising cities: the example of advertising firms in Stockholm. The Service Industries Journal.

[CR11] Hertog P, Aa W, Jong M (2010). Capabilities for managing service innovation: towards a conceptual framework. Journal of Service Management.

[CR12] Hipp C, Grupp H (2005). Innovation in the service sector: The demand for service-specific innovation measurement concepts and typologies. Res Policy.

[CR13] Hsu CW, Hu A, Chiou C, Chen TC (2011). Using the FDM and ANP to construct a sustainability balanced scorecard for the semiconductor industry. Expert Syst Appl.

[CR14] Hult G, Snow C, Kandermir D (2003). The Role of Entrepreneurship in Building Cultural Competitiveness in Different Organizational Types. J Manag.

[CR15] Jalali Naini SG, Aliahmadia AR, Jafari-Eskandaria M (2010). Designing a mixed performance measurement system for environmental supply chain management using evolutionary game theory and Balanced Scorecard: A case study of an auto industry supply chain. Resour Conserv Recycl.

[CR16] Jeffcutt P, Pratt AC (2002). Managing Creativity in the Cultural Industries. Creativity and Innovation Management.

[CR17] Kaplan R, Norton D (2000). Cuadro de Mando Integral.

[CR18] Keeney R, Raiffa H (1976). Decisions with multiple objectives: preferences and values tradeoffs.

[CR19] Ko H-T, Lu H-P (2010). Measuring innovation competencies for integrated services in the communications industry. Journal of Service Management.

[CR20] Lampel J, Lant T, Shamsie J (2000). Balancing act: Learning from organizing practices in cultural industries. Organ Sci.

[CR21] Lee A, Chen H, Tong Y (2008a). Developing new products in a network with efficiency and innovation. Int J Prod Res.

[CR22] Lee A, Chen W-C, Chang C-J (2008b). A Fuzzy AHP and BSC approach for evaluating performance of IT department in the manufacturing industry in Taiwan. Expert Syst Appl.

[CR23] Leung LC, Lam KC, Cao D (2006). Implementing the balanced scorecard using the analytic hierarchy process and the analytic network process. J Oper Res Soc.

[CR24] Liedtka S (2005). Analytic Hierarchy Process and multi-criteria performance management systems. Cost Management.

[CR25] Lightfoot HW, Gebauer H (2011). Exploring the alignment between service strategy and service innovation. Journal of Service Management.

[CR26] Lin Y-H, Tsai K-M, Shiang W-J, Kuo T-C, Tsai C-H (2009). Research on using ANP to establish a performance assessment model for business intelligence systems. Expert Syst Appl.

[CR27] Nachum L (1996). Winners and losers in professional service industries: What makes the difference?. The Service Industries Journal.

[CR28] Öztayşi B, Uçal Ĭ (2009). Comparing MADM Techniques For Use In Performance Measurement. ISAHP 2009 Proceedings. The 10th International Symposium on the Analytic Hierarchy Process. July 29- August 1, 2009. Pittsburgh, PA, USA.

[CR29] Öztayşi B, Kaya T, Kahraman C (2011). Performance comparison based on customer relationship management using analytic network process. Expert Syst Appl.

[CR30] Porter M (1995). Ventaja Competitiva.

[CR31] Porter M (1997). Estrategia Competitiva.

[CR32] Poveda-Bautista R, Baptista D, García-Melón M (2012). Setting competitiveness indicators using BSC and ANP. Int J Prod Res.

[CR33] Pratt AC (2006). Advertising and creativity, a governance approach: a case study of creative agencies in London. Environment and Planning A.

[CR34] Raisinghani HS, Meade L, Schkade L (2007). Strategic e-business decision analysis using Analytic Network Process. IEEE transactions on Engineering Management.

[CR35] Reisinger H, Cravens K, Tell N (2003). Prioritizing performance measures within the Balanced Scorecard framework. Manag Int Rev.

[CR36] Ren Q’e, Gong X (2012). Evaluation Index System for Academic Papers of Humanities and Social Sciences. Scientometrics.

[CR37] Roy S (2011). Competitiveness in Service Sector: A case of Hotel Industry in India. Glob Bus Rev.

[CR38] Saaty T (1980). The Analytic Hierarchy Process.

[CR39] Saaty T (1996). The Analytic Hierarchy Process: planning, priority setting, resource allocation.

[CR40] Saaty T (2000). Fundamentals of Decision Making and priority theory with the Analytic Hierarchy Process.

[CR41] Saaty T (2005). Theory and Applications of the Analytic Network Process.

[CR42] Shahin A, Mahbod MA (2007). Prioritization of key performance indicators: An integration of analytical hierarchy process and goal setting. Int J Product Perform Manag.

[CR43] Sigala M (2012). Social networks and customer involvement in New Service Development (NSD. Int J Contemp Hosp Manag.

[CR44] Sirikrai S, Tang J (2006). Industrial competitiveness analysis: Using the analytic hierarchy process. Journal of High Technology Management Research.

[CR45] Smith-Perera A, García-Melón M, Poveda-Bautista R, Pastor-Ferrando J (2010). A Project Strategic Index proposal for portfolio selection in electrical company based on the Analytic Network Process. Renew Sustain Energy Rev.

[CR46] Spendolini M (1994). Benchmarking.

[CR47] (2009). CREATIVE decision Foundation.

[CR48] Taylor PJ (2006). Advertising and Cities: a relational geography of Globalization in the early Twenty First Century. GaWC Research Bulletin, 215.

[CR49] Taylor R, Grubbs H, Haley E (1996). How French advertising professionals develop creative strategy. The journal of advertising.

[CR50] Temur G, Emeksizoglu B, Gozlu S (2007). A Study on performance measurement of a Plastic Packaging Organization’s Manufacturing System by AHP Modeling. PICMET 2007 Proceedings.

[CR51] Thakkar J, Deshmukh SG, Gupta AD, Shankar R (2007). Development of a balanced scorecard. An integrated approach of interpretative Structural Modeling (ISM) and Analytic Network Process(ANP). Int J Product Perform Manag.

[CR52] Tseng M-L (2010). Implementation and performance evaluation using the fuzzy network balanced scorecard. Computers and Education.

[CR53] Yang C-L, Chuang S-P, Huang R-H (2009). Manufacturing evaluation system based on AHP/ANP approach for wafer fabricating industry. Expert Syst Appl.

[CR54] Yüksel I, Dagdeviren M (2010). Using the fuzzy analytic network process (ANP) for Balanced Scorecard (BSC): A case study for manufacturing firm. Expert Syst Appl.

